# Glucose Metabolism Disorder Induces Spermatogenic Dysfunction in Northern Pig-Tailed Macaques (*Macaca leonina*) With Long-Term SIVmac239 Infection

**DOI:** 10.3389/fendo.2021.745984

**Published:** 2021-09-24

**Authors:** Tian-Zhang Song, Ming-Xu Zhang, Han-Dan Zhang, Xue-Hui Wang, Wei Pang, Ren-Rong Tian, Yong-Tang Zheng

**Affiliations:** ^1^ Key Laboratory of Animal Models and Human Disease Mechanisms of the Chinese Academy of Sciences, Kunming Institute of Zoology - the Chinese University of Hong Kong (KIZ-CUHK) Joint Laboratory of Bioresources and Molecular Research in Common Diseases, Center for Biosafety Mega-Science, Kunming Institute of Zoology, Chinese Academy of Sciences, Kunming, China; ^2^ School of Life Sciences, University of Science and Technology of China, Hefei, China; ^3^ National Resource Center for Non-Human Primates, National Research Facility for Phenotypic & Genetic Analysis of Model Animals (Primate Facility), Kunming Institute of Zoology, Chinese Academy of Sciences, Kunming, China

**Keywords:** spermatogenic dysfunction, glucose metabolism, *Macaca leonina*, northern pig-tailed macaques, SIVmac239

## Abstract

Although spermatogenic dysfunction is widely found in patients with human immunodeficiency virus (HIV), the underlying reasons remain unclear. Thus far, potential hypotheses involving viral reservoirs, testicular inflammation, hormone imbalance, and cachexia show inconsistent correlation with spermatogenic dysfunction. Here, northern pig-tailed macaques (NPMs) exhibited marked spermatogenic dysfunction after long-term infection with simian immunodeficiency virus (SIVmac239), with significant decreases in Johnsen scores, differentiated spermatogonial stem cells, and testicular proliferating cells. The above hypotheses were also evaluated. Results showed no differences between SIV− and SIV+ NPMs, except for an increase in follicle stimulating hormone (FSH) during SIV infection, which had no direct effect on the testes. However, long-term SIVmac239 infection undermined pancreatic islet β cell function, partly represented by significant reductions in cellular counts and autophagy levels. Pancreatic islet β cell dysfunction led to glucose metabolism disorder at the whole-body level, which inhibited lactate production by Sertoli cells in testicular tissue. As lactate is the main energy substrate for developing germ cells, its decrease was strongly correlated with spermatogenic dysfunction. Therefore, glucose metabolism disorder appears to be a primary cause of spermatogenic dysfunction in NPMs with long-term SIVmac239 infection.

## Introduction

Histological changes in the testes of autopsied patients with acquired immunodeficiency syndrome (AIDS) were first noted in 1987, with 62.5% of cases showing hypospermatogenesis (1). Since then, nearly 200 autopsy studies have confirmed that spermatogenesis is significantly reduced in AIDS patients. Furthermore, atypical testis lesions (e.g., interstitial inflammation and fibrosis and tubular basement membrane thickening) are found in many cases (2–4). However, the causes of spermatogenic dysfunction in patients with human immunodeficiency virus (HIV) remain unclear.

Five main hypotheses for spermatogenic dysfunction during HIV infection have been explored. (A) Viral reservoir in testes:> Several studies have identified HIV RNA in the semen (range, 4%–48%) of chronically infected patients receiving combination antiretroviral therapy (cART) (5). However, the release of viruses in semen is a complex process, involving the seminal vesicles, epididymis, and vas deferens (6–8). Previous research on HIV sequences suggests that testicular viruses originate from peripheral blood and do not contaminate the seminiferous epithelium (6). In addition, while SIV/SHIV can infect spermatogonia in juvenile macaques (9), immunohistochemical analyses suggest that only immune cells located within the interstitial tissues are target cells of HIV/SIV (10, 11). (B) Testicular inflammation: Infection and inflammation of the male genital tract can damage immune tolerance and induce male infertility (12). Immune cells located within the interstitial tissues include central and effector memory CD4+ and CD8+ T cells, as well as macrophages, dendritic cells (DCs), and natural killer (NK) cells (13). However, although interstitial inflammation is reported in HIV patients (2–4), its incidence is significantly lower than that of reduced spermatogenesis (20%–30% *vs >*70%) (1–4). In addition, although SIV infection can cause changes in testicular T cell phenotypes and macrophage and DC counts (14), compared with seminal vesicles, the immune microenvironment in the testis is stable and pro-inflammatory factors show no relationship with semen viral load during chronic SIVmac251 infection (6). (C) Hormone imbalance: Sex hormones, including follicle stimulating hormone (FSH), luteinizing hormone (LH), and testosterone, are strongly connected to testis development and spermatogenic function (15, 16). In AIDS patients, low levels of serum testosterone induced by hypothalamic-pituitary dysfunction are the most common cause of hypogonadism (17). Compared with uninfected individuals, however, the concentrations of FSH, LH, and testosterone in serum are reported to be unchanged in non-progressors (18). (D) Cachexia: Cachexia is a state of general ill health and malnutrition characterized by excessive weight loss and accelerated skeletal muscle loss (19, 20). In HIV-infected patients, those with cachexia show three times higher rates of testicular dysfunction than those without (21). Cachexia can also lead to azoospermia in rhesus macaques with AIDS (22), however testicular dysfunction also exists in SIV-infected macaques without cachexia (14). (E) Glucose metabolism disorders: Based on clinical cross-sectional research, HIV-infected patients can exhibit glucose metabolism disorder and pancreatic islet β cell dysfunction before cART (23). Epidemiological studies have also shown that nearly 50% of diabetic patients suffer from various reproductive system diseases (24–26), such as decreased libido, impotence, erectile dysfunction, ejaculation difficulties, reduced sperm quality, and infertility (27–29). Energy metabolism is a key factor supporting spermatogenesis, including cell proliferation, meiotic division, and differentiation of post-meiotic cells into spermatozoa. In mammals, this process occurs under the influence of Sertoli cells, which “nurse” spermatogenic cells by releasing lactate as an end product of glycolytic metabolism (30). Lactate is taken up and metabolized by meiotic and post-meiotic spermatogenic cell mitochondria. In round spermatids, external lactate is an efficient metabolite for oxidative metabolism in these cells. However, the relationship between glucose metabolism disorders and spermatogenic dysfunction in HIV-infected patients or SIV-infected macaques remains unclear.

Pig-tailed macaques are valuable HIV/AIDS animal models. We previously identified northern pig-tailed macaques (NPMs, *Macaca leonina*) as suitable long-term HIV- and SIV-infected animal models for AIDS research (31–34). Although both rhesus macaques and pig-tailed macaques present obvious pathological changes in the testes similar to human patients with AIDS (14, 35), the utility of rhesus macaques is limited due to their obvious seasonality in endocrine and exocrine testicular function (36). Therefore, in the current study, we examined histological changes in the testes of NPMs with chronic SIVmac239 infection and then explored and evaluated the above hypotheses to identify potential causes of spermatogenic dysfunction.

## Materials and Methods

### Animals and Ethics Statement

Eight adult male NPMs (6–8 years old), negative for SIV, B virus, simian type-D retrovirus, and simian T-lymphotropic virus, were enrolled in this study. The characteristics of the enrolled macaques have been discussed in our previous studies (33, 34, 37). Briefly, four NPMs (named 08287, 09203, 10205, and 10225) were intravenously injected with 3 000 TCID_50_ SIVmac239 and euthanized on days 620, 633, 642, and 650 post-injections, respectively. The other four NPMs (08269, 09223, 09237, and 09255) without SIVmac239 inoculation were regarded as the SIV− group. All animal experiments and procedures were approved by the Ethics Committee of the Kunming Institute of Zoology, Chinese Academy of Sciences (approval number: SYDW-2015023).

### Study Design

After 12 h of fasting, the monkeys were anesthetized with ketamine hydrochloride. Subsequently, peripheral blood was collected through venipuncture into vacuum tubes containing ethylenediaminetetraacetic acid (EDTA) for hormone and lipo-metabolic index analyses on days 0, 3, and 5 before euthanasia. Testicular tissues and pancreatic tails were collected within 5 min of euthanasia, which was induced by an excessive dose of ketamine hydrochloride. Isolated tissues were washed in phosphate-buffered saline (PBS) and then fixed in 4% polyformaldehyde for histological examination and immunostaining. The remaining testes were cut into pieces for tissue RNA extraction, interstitial cell isolation, and homogenized tissue preparation.

### Viral RNA Detection in Plasma and Testes

Total RNA was extracted from plasma using a High Pure Viral RNA Kit (Roche, UK) following the manufacturer’s instructions (33, 34). Testicular tissues were homogenized, and tissue RNA was extracted using TRIzol Reagent (Thermo, USA). For detection of SIVmac239 RNA, a THUNDERBIRD Probe One-Step qRT-PCR Kit (TOYOBO, Japan) was used following the manufacturer’s protocols. Primers and probes included: forward primer 5’-TCGGTCTTAGCTCCATTAGTGCC-3’; reverse primer 5’-GCTTCCTCAGTGTGTTTCACTTTC-3’; and probe 5’-CTTCTGCGTGAA TGCACCAGATGACGC-3’.

### Expression of Cytokines in Testes

Tissue RNA extracted from testes was reverse transcribed using a PrimeScript RT Reagent Kit (TAKARA, Japan). Gene expression was measured using SYBR Green quantitative polymerase chain reaction (qPCR), as described previously (38). In brief, the cDNA of 16 genes involved in inflammatory response were amplified with SYBR Premix Ex Taq II (TAKARA, Japan) in a ViiA7 Real-Time PCR System (Thermo, USA) and normalized to GAPDH expression determined in parallel real-time (RT) PCR reactions. Relative gene expression was expressed as -ΔCt, which represents the difference between the Ct values of GAPDH and the target gene. Primers used are summarized in **Supplementary Table 1**.

### Flow Cytometry

Immune cells in peripheral blood were collected and counts and phenotypes were analyzed by flow cytometry. The original data are reported in our previous studies (33, 34). Interstitial cells in the testes were isolated as described previously (10). Briefly, testicular tissues were chopped and then incubated at 37°C with collagenase and DNase for 1 h. Tubule fragments were allowed to settle for 3 min, followed by interstitial cell recovery in PBS. Subsequently, the phenotypes of the interstitial cells were analyzed by multi-parameter flow cytometry. The anti-human flow cytometry antibodies cross-reactive with NPMs included anti-CD45-PE (557059, BD, USA), anti-CD3-APC-Cy7 (557757, BD), anti-CD20-FITC (302304, BioLegend, USA), anti-CD4-PerCP-Cy5.5 (317428, BioLegend), anti-CD8-PE-Cy7 (557746, BD), anti-Ki67-PE (51-36525X, BD), and anti-PD-1-PE (329906, BioLegend).

### Biochemical Indices

The concentrations of glucose, testosterone, triglycerides, cholesterol, low-density lipoprotein (LDL), and high-density lipoprotein (HDL) in plasma as well as testosterone in homogenized testes were detected using an automatic biochemical analyzer (Cobas8000, Roche, Germany). The levels of insulin and c-peptide in plasma were tested using a chemiluminescence instrument (E601, Roche, Germany). In addition, the contents of FSH and LH in plasma were assessed using corresponding enzyme-linked immunosorbent assay (ELISA) kits (YuTong, China) in accordance with the manufacturer’s instructions. The concentration of lactate in the homogenized testes was detected using a LD ELISA kit (Jiancheng, China) in accordance with the manufacturer’s instructions.

### Histological and Immunological Staining

Fixed testicular tissues were cut into 4-μm-thick sections for hematoxylin-eosin (H&E) staining. Histological changes in seminiferous tubules were analyzed and scored (**Supplementary Table 2**) following previous research (39). Immunological staining was performed as per Zhang et al. (32), with some modification. Rehydrated sections were processed at high pressure for 3 min in 1% citraconic anhydride (pH 9.0) instead of microwave heating in saline sodium citrate buffer. Primary antibodies were diluted in bovine serum albumin (BSA) and incubated at room temperature for 120 min. The sections were then washed and incubated with secondary antibodies in BSA for 60 min at room temperature. Primary antibodies included rabbit anti-p27 polyclonal antibodies generated in our laboratory, mouse anti-insulin antibody (NBP2-45108, NOVUS, USA), rabbit anti-glucagon antibody (ab133195, Abcam, England), rabbit anti-LDHA antibody (ab84716, Abcam), rabbit anti-AMH antibody (ab84952, Abcam), mouse anti-SOX9 antibody (ab76997, Abcam), sheep anti-GLUT1 antibody (ab54263, Abcam), rabbit anti-MCT4 antibody (ab180699, Abcam), rabbit anti-c-kit antibody (ab32363, Abcam), mouse anti-PCNA antibody (ab29, Abcam), rabbit anti-LC3B antibody (ab63817, Abcam), rabbit anti-CD45 antibody (ab10558, Abcam), and rabbit anti-cleaved caspase-3 antibody (#9664, Cell Signaling, USA). Secondary antibodies included donkey anti-rabbit IgG antibody (ab181346, Alexa Fluor^®^ 488, Abcam), donkey anti-rabbit IgG antibody (ab150070, Alexa Fluor^®^ 555, Abcam), donkey anti-mouse IgG antibody (ab150106, Alexa Fluor^®^ 555, Abcam), goat anti-mouse IgG antibody (A11001, Alexa Fluor^®^ 488, Life Technologies, USA), donkey anti-sheep IgG antibody (ab150178, Alexa Fluor^®^ 555, Abcam), and donkey anti-rabbit IgG antibody (ab6802, HRP, Abcam).

### Gene Set Enrichment Analysis

The transcription profile dataset of testicular tissues from individuals with glucose metabolism disorder was searched in the NCBI GEO database (https://www.ncbi.nlm.nih.gov/geo/). Only one dataset matched, which included three adult mice (4 months old) fed a standard diet and three adult mice (4 months old) fed a high-fat diet (accession number GSE44301) (40). The platform used for GSE44301 was GPL6887 (Illumina MouseWG-6 v2.0 Expression BeadChip, Illumina Inc., San Diego, CA, USA). In this study, the R (v4.0.2) and Bioconductor packages were used for data mining and statistical analyses. The GEOquery package was used to download the microarray data. The Limma package was used for the calculation of aberrantly expressed mRNAs. Differentially expressed genes (DEGs) between diabetic and control groups were identified with cut-off criteria of *P* < 0.05 and absolute log_2_FC > 0.58. The GSEABase package was used to analyze significant gene sets.

### Statistical Analysis

All data are presented as mean ± standard deviation (SD). The Pearson rank test was used to determine correlations between two datasets. The Mann-Whitney nonparametric test was used to analyze differences between SIV+ and SIV− groups. All statistical analyses were performed using GraphPad Prism (v8, GraphPad Software, USA). *P* < 0.05 was considered statistically significant.

## Results

### Continuous Disease Progression During SIVmac239 Infection

**Figure 1:**
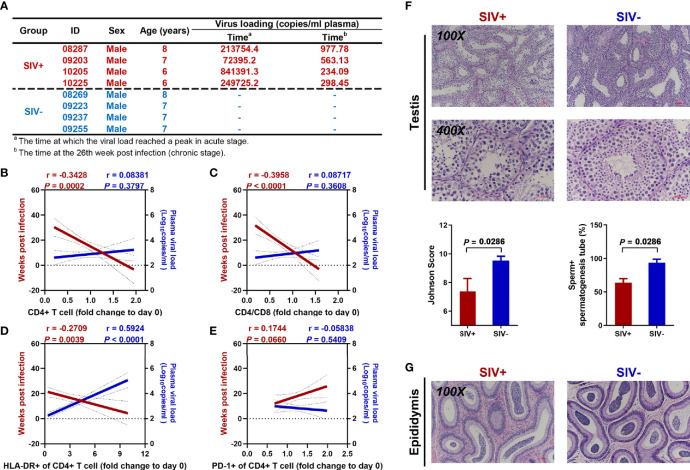
Continuous disease progression and spermatogenic dysfunction in NPMs with long-term SIVmac239 infection. **(A)** Characteristics of enrolled macaques; **(B)** Correlation analysis of CD4+ T cell count with duration of infection and viral load in plasma; **(C)** Correlation analysis of CD4+/CD8+ cell ratio with duration of infection and viral load in plasma; **(D)** Correlation analysis of proportion of CD4+ T cells expressing HLA-DR with duration of infection and viral load in plasma; **(E)** Correlation analysis of proportion of CD4+ T cells expressing PD-1 with duration of infection and viral load in plasma; **(F)** Testicular H&E staining and pathological analysis; **(G)** Epididymal H&E staining.

Long-term observations were performed on the NPMs injected with SIVmac239. The plasma PCR results showed a peak viral load 2–4 weeks post-injection, with a lower load during the chronic stage than during the acute stage (**Figure 1A**). Before sacrifice, NPMs 09203, 10205, and 10225 showed negative viral RNA detection in plasma, while NPM 08287 showed a positive viral load of 1 162.7 copies/mL. Compared with the plasma viral load, duration of infection showed a strong correlation with changes in CD4+ T cells in the blood. Although the plasma viral load gradually decreased during the chronic stage, long-term infection induced significant decreases in the CD4+ T cell count (**Figure 1B**), CD4+ to CD8+ T cell ratio (**Figure 1C**), and activated CD4+ T cell count (defined by HLA-DR expression) (**Figure 1D**), as well as an increase in exhausted CD4+ T cell count (defined by PD-1 expression) (**Figure 1E**).

### Spermatogenic Dysfunction and Hypothesis Identification

Compared with the SIV− group, long-term SIVmac239 infection undermined spermatogenic function in the NPMs, with a significant decrease in the Johnson score (6 to 8) and percentage of tubules with sperm (50% to 75%) (**Figure 1F**). However, partial spermatogenic function was retained as sperm could be found in the epididymides of both SIV− and SIV+ NPMs (**Figure 1G**).

**Figure 2:**
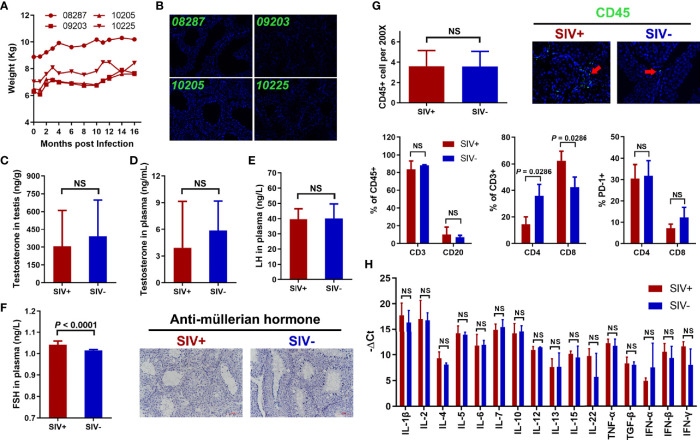
Verification of hypotheses for spermatogenic dysfunction in NPMs with long-term SIVmac239 infection. **(A)** Changes in body weight during SIV infection; **(B)** Immunological staining of p27 in testicular tissues; **(C)** Concentration of testosterone in testis; **(D)** Concentration of testosterone in plasma; **(E)** Concentration of LH in plasma; **(F)** Concentration of FSH in plasma and immunological staining of AMH; **(G)** Immune cell count and phenotype in testicular tissue; **(H)** Expression levels of cytokines in testicular tissue. NS, no statistic significance.

**Figure 3:**
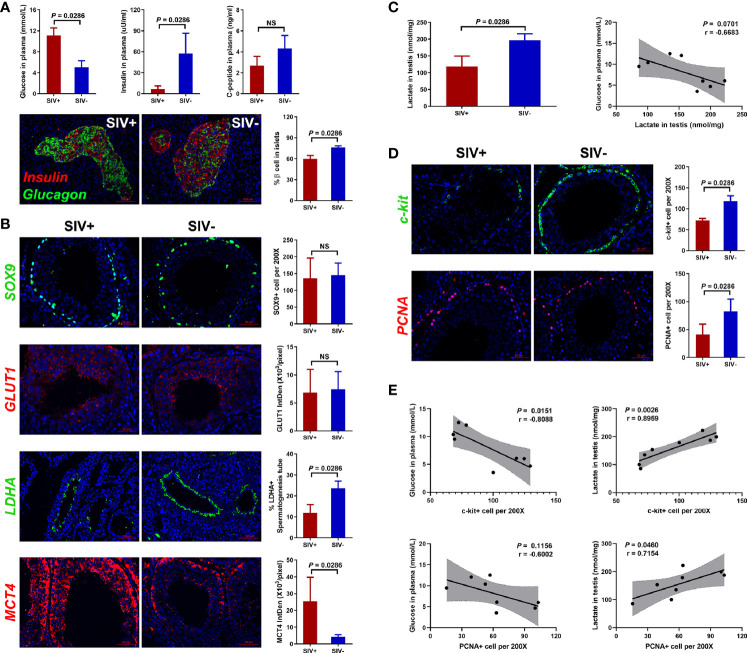
Glucose metabolism disorder and related testicular changes. **(A)** Concentrations of glucose, insulin, and c-peptide in plasma, and immunological staining of insulin and glucagon in pancreatic islets; **(B)** Glucose metabolism analysis of Sertoli cells in testicular tissues; **(C)** Lactate level in testicular tissues; **(D)** Immunological staining of c-kit and PCNA in testicular tissues; **(E)** Correlation analysis of c-kit+/PCNA+ cells with glucose in plasma and lactate in testes. NS, no statistic significance.

The abovementioned hypotheses were examined, with the following results: Cachexia: SIVmac239 infection did not induce obvious weight loss in the NPMs in either the acute or chronic stages (**Figure 2A**). Viral reservoir: Based on PCR and immunofluorescence, no viral RNA (data not plotted) or Gag protein was found in the testicular tissues (**Figure 2B**). Sex hormones: The concentrations of testosterone in testicular tissue (**Figure 2C**) as well as testosterone and LH in plasma (**Figure 2D, E**) showed no differences between SIV− and SIV+ NPMs. However, the level of FSH was markedly increased in the SIV+ NPMs compared to the SIV− group (**Figure 2F**). The primary role of FSH in spermatogenesis is the stimulation of Sertoli cell proliferation and the increase in anti-Müllerian hormone (AMH) expression and transcription (41, 42). Here, AMH expression levels (**Figure 2F**) and Sertoli cell counts (**Figure 3B**) were analyzed by immunohistochemical staining. However, no differences were detected between the SIV− and SIV+ NPMs, implying that FSH may have no direct effects on spermatogenic function in NPMs. Inflammation: Immune cells in testicular tissues (defined by CD45 expression) were only identified in the interstitial areas. No infiltration into tubules or amplification of immune cells was found during infection (**Figure 2G**). The CD3+ and CD20+ cell rates remained almost the same between the two groups. Although SIVmac239 infection changed the composition of T cells, with decreased CD4+ T cells and increased CD8+ T cells, the activation (defined by HLA-DR expression, data not plotted) and exhaustion (defined by PD-1 expression) of CD4+ T cells and CD8+ T cells were very similar between the SIV− and SIV+ NPMs (**Figure 2G**). Based on cytokine expression, no differences were observed in the testis-immune microenvironment between the SIV− and SIV+ NPMs (**Figure 2H**).

### Glucose Metabolism Disorder Induced Spermatogenic Dysfunction

SIVmac239 infection induced obvious glucose metabolism disorder, as seen by the decrease in insulin and c-peptide in plasma and increase in glucose (**Figure 3A**). We also calculated the percentage of pancreatic α cells (defined by glucagon expression) and β cells (defined by insulin expression) in pancreatic islets and found that pancreatic β cells decreased markedly in the SIVmac239-infected NPMs (**Figure 3A**).

Energy metabolism in the testes is highly dependent on Sertoli cells, which absorb glucose in plasma, transform it to lactate, and provide it to cells inside the blood-testis barrier as energy (43). Here, we detected no differences in Sertoli cell count (defined by SOX9 expression) or glucose transporter expression (defined by GLUT1 expression) between the SIV− and SIV+ NPMs (**Figure 3B**). However, the expression of lactate dehydrogenase A (LDHA), in response to the conversion of pyruvate to lactate, was detected by immunofluorescence and the ratio of LDHA+ seminiferous tubules was significantly reduced in SIV+ NPMs. In addition, the expression of mono-carboxylate transporter 4 (MCT4), which transports lactate, was significantly increased during SIVmac239 infection. These changes in Sertoli cells suggested the occurrence of lactate metabolism disorder during infection. Therefore, the concentration of lactate in testicular tissues was assessed by ELISA, which showed an obvious decrease in the SIV+ NPMs (**Figure 3C**). In addition, a weak negative correlation was found between the level of glucose in plasma and the concentration of testicular lactate (**Figure 3C**). Spermatogenesis starts in spermatogonial stem cells (SSCs). Once SSCs have committed to differentiate, the subsequent cellular processes progress in a strictly ordered manner up to sperm formation in the seminiferous tubules (44). Compared with the SIV− group, long-term SIVmac239 infection induced obvious decreases in the number of differentiated SSCs (defined by c-kit expression) and proliferating cells (defined by PCNA expression) (**Figure 3D**). In addition, the number of c-kit+ and PCNA+ cells showed strong positive correlations with the concentration of testicular lactate and relatively weak negative correlations with the level of glucose in plasma, indicating that lactate metabolism disorder causes spermatogenic dysfunction in NPMs with long-term SIVmac239 infection (**Figure 3E**).

**Figure 4:**
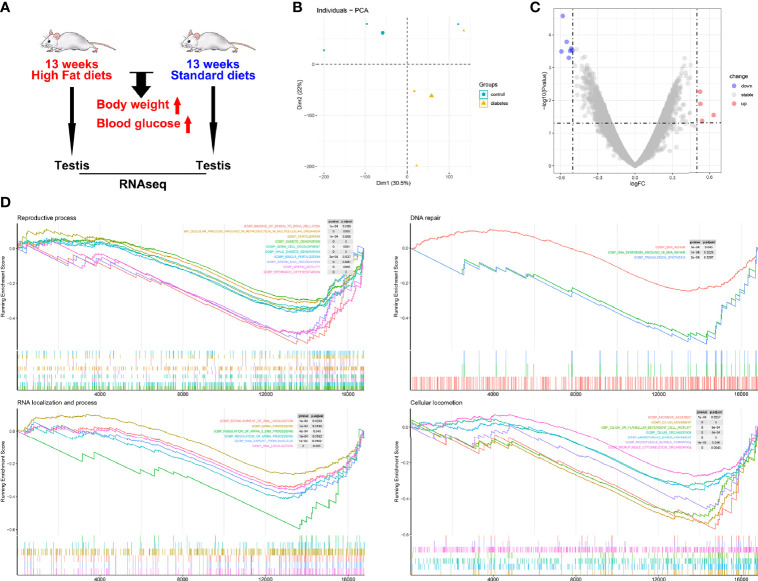
Spermatogenic dysfunction in diabetic mice. **(A)** Characteristics of enrolled animals in GSE44301; **(B)** Principal component analysis; **(C)** Differentially expressed genes (DEGs) between diabetic and control mice; **(D)** Gene set enrichment analysis.

As plasma glucose showed a relatively weak relationship with testicular lactate and spermatogenic function, we analyzed data from the GEO database to confirm the direct effects of glucose metabolism on spermatogenic function. Testicular gene transcription profiles were compared between diabetic mice (induced by a high-fat diet) and control mice (fed a standard diet) (**Figure 4A**). Principal component analysis (PCA) showed significant differences in the expression profiles of the two groups (**Figure 4B**). As only 11 DEGs (cut-off of *P* < 0.05 and absolute log2FC > 0.50) were identified between diabetic and control mice (**Figure 4C**), we conducted GSEA to identify gene sets associated with spermatogenic function. Ten gene sets related to reproductive processes, three gene sets related to DNA repair, six gene sets related to RNA localization and processes, and seven gene sets related to cellular locomotion showed obvious differences between the diabetic and control mice (**Figure 4D**). Compared with the control group, the diabetic mice showed lower expression of gene sets related to spermatogenic function, thus proving the direct effects of glucose metabolism on spermatogenic function.

### SIV Infection Inhibited Pancreatic Islet β Cell Autophagy

**Figure 5:**
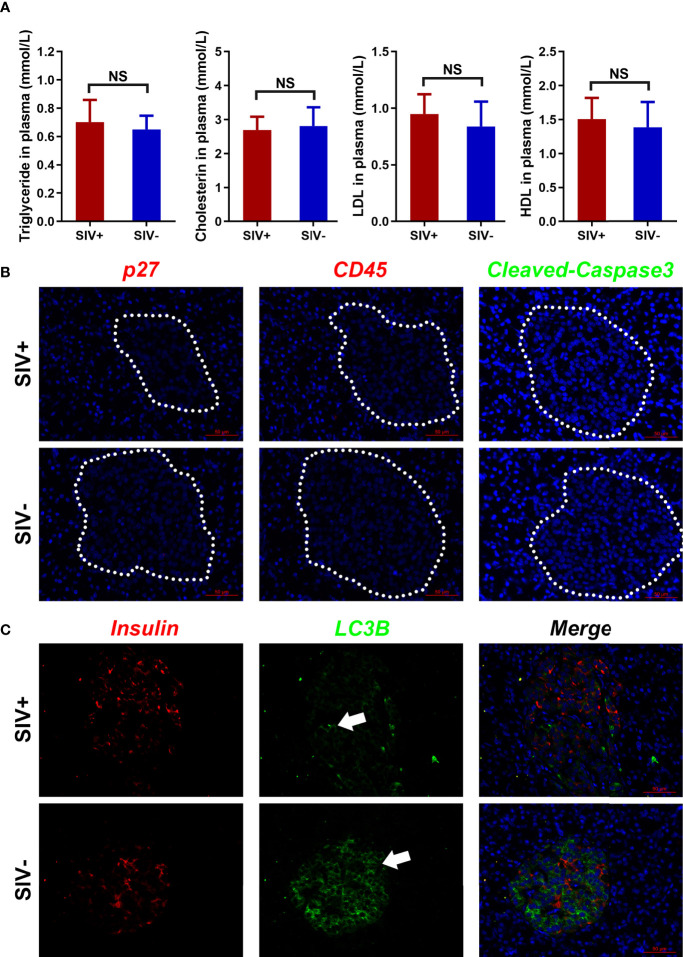
Lipid metabolism and pathological changes in pancreatic islets. **(A)** Index analysis of lipid metabolism (triglycerides, cholesterin, LDL, and HDL); **(B)** Immunological staining of p27, CD45, and cleaved-caspase3 in pancreatic islets; **(C)** Immunological staining of insulin and LC3B in pancreatic islets. NS, no statistic significance.

In the abovementioned GEO dataset, the long-term high-fat diet led to glucose metabolism disorder. Therefore, we assessed the levels of triglycerides, cholesterol, LDL, and HDL in the plasma of SIV− and SIV+ NPMs and found no significant differences (**Figure 5A**). In addition, viral infection, immune infiltration, and cellular apoptosis (defined by p27, CD45, and cleaved-caspase3, respectively) were not detected in the pancreatic islets of SIV+ NPMs (**Figure 5B**). However, compared with SIV− NPMs, a markedly lower level of autophagy (defined by LC3B expression) was detected in the pancreatic islet β cells of NPMs with long-term SIVmac239 infection (**Figure 5C**). Autophagy is necessary to maintain the structure, mass, and function of pancreatic islet β cells. Thus, impaired autophagy may explain the pancreatic islet β cell damage detected during chronic SIVmac239 infection. However, the reasons for reduced autophagy in pancreatic islet β cells in SIV+ NPMs remain unclear.

## Discussion

NPMs are valuable HIV/AIDS animal models (31–34). By analyzing the correlation between CD4+ T cells and duration of infection, SIVmac239-infected NPMs showed continuous disease progression in the chronic stage. Although sperm was visible in the epididymis, long-term SIVmac239 infection induced obvious pathological changes in the testicular tissue of NPMs, leading to significant decreases in differentiated SSCs and proliferating cells. We also explored previous hypotheses related to spermatogenic dysfunction in patients with HIV/AIDS. However, we detected no pathological features of cachexia, testicular viral reservoirs, or testicular inflammation in the SIV+ NPMs, and increased FSH showed no direct effects on spermatogenic function. In addition, plasma viral load was negative in three NPMs (09203, 10205, and 10225) but was detectable in 08287 at the stage of euthanasia. We also found long-term positive plasma viral load in a NPM (08247) in our previous study (34), which may be due to individual differences during SIVmac239 infection. However, all enrolled NPMs with SIV infection showed consistent changes in immune cell phenotypes, testicular pathology, and pancreatic injury. In addition, no viral infiltration was detected in the testes or pancreatic islets of the NPMs with positive or negative plasma viral loads, suggesting viral particles were not directly responsible for spermatogenic dysfunction. These results strongly imply the existence of a yet-to-be-discovered mechanism underlying spermatogenic dysfunction in NPMs. Interestingly, upon further experimentation, we confirmed that glucose metabolism disorder was the main cause of spermatogenic dysfunction in NPMs with long-term SIVmac239 infection.

The long-term utility of cART in HIV patients is associated with multiple metabolic complications, including abnormal blood lipids, insulin resistance, and metabolic syndrome (45–47). HIV infection can directly influence glucose metabolism in antiretroviral-naïve patients. Comparing 50 HIV patients not receiving cART with 50 volunteers, Lan et al. found that the plasma concentration of glucose is increased in HIV infectors. Moreover, HIV infection significantly inhibits the homeostasis model assessment of insulin resistance (HOMA-IR) and homeostasis model assessment of β cell (HOMA-β) indices in untreated patients (23). These pathological features have been confirmed *via* cross-sectional analysis of a longitudinal cohort of 710 untreated patients with HIV and 226 volunteers (48). Notably, compared with uninfected individuals, the plasma concentration of insulin and the HOMA-IR index are significantly decreased in HIV patients receiving no cART. In the current study, glucose metabolism disorder, including increased glucose concentration and decreased insulin and c-peptide levels, was detected in the NPMs following long-term SIVmac239 infection, similar to antiretroviral-naïve patients with HIV infection.

Reproductive dysfunction is a major secondary complication in both diabetic animals and humans (49). Gonadosomatic indices and relative weights of testes, epididymides, and seminal vesicles all significantly decrease in streptozotocin-induced animal models of diabetes (50). In addition, pathological evidence clearly shows malignant histopathological changes in the seminiferous tubules of diabetic animals, including a reduction in seminiferous tubule size and degeneration and vacuolization of spermatogonia, spermatocytes, and spermatids (51). In this study, we analyzed data from the GEO database to confirm the direct effects of glucose metabolism on spermatogenic function. Gene sets related to reproductive processes, DNA repair, RNA localization and processes, and cellular locomotion were all markedly decreased in the diabetic mice compared with the control mice. Glucose metabolic cooperation between Sertoli cells and developing germ cells is one of the most important events during spermatogenesis (43, 52). Sertoli cells demonstrate high glycolytic flux to ensure the production of lactate and factors required for developing germ cells (53, 54). Previous research has shown that the testicular concentration of lactate is lower in diabetic rats with decreased LDH activity and increased MCT4 expression (55). In this study, we found that the testicular concentration of lactate was substantially decreased during SIVmac239 infection and the Sertoli cells in SIV+ NPMs showed similar changes as observed in diabetic rats. In addition, significant negative correlations were found between the concentrations of lactate in the testes and differentiated SSCs and proliferating cells. These results suggested that glucose metabolism disorder caused spermatogenic dysfunction in NPMs with long-term SIVmac239 infection.

The changed levels of glucose, insulin, and c-peptide in the SIV+ NPMs implied that pancreatic islet β cell dysfunction may be the starting point of glucose metabolism disorder during SIVmac239 infection. However, glucose metabolism disorder was regarded as a non-essential reason for spermatogenic dysfunction during SIV infection at the design phase of this study due to limited evidence in untreated HIV patients from previous studies. Therefore, pancreatic islets were not isolated from the pancreas during euthanasia. Using fixed tissues, however, the number of pancreatic islet β cells decreased significantly in the NPMs with long-term SIVmac239 infection. Moreover, compared with the SIV− NPMs, a markedly lower level of autophagy (defined by LC3B expression) was detected in the pancreatic islet β cells of SIV+ NPMs, whereas viral infection, immune infiltration, and cellular apoptosis were not detected in the islets. Clear evidence for the role of impaired autophagy in pancreatic islet β cell dysfunction has been reported in mice following β cell-specific knockout of Atg7 (56, 57). Autophagy is necessary to maintain the structure, mass, and function of pancreatic islet β cells, and its impairment can cause insulin deficiency and hyperglycemia due to abnormal turnover and function of cellular organelles (57). Therefore, impaired autophagy may be a reason for pancreatic islet β cell damage during chronic SIVmac239 infection. However, due to pancreatic sample limitations, the characteristics of the immune microenvironment, viral infection, and pancreatic islet β cells as well as the reasons for reduced autophagy of pancreatic islet β cells in SIV+ NPMs remain uncertain.

We previously detected obvious microbial translocation and immune activation in NPMs with SIVmac239 infection (37). Notably, average concentrations of lipopolysaccharide (LPS)-binding protein (a microbial translocation marker) in plasma increased from 12 to 36 weeks post-SIVmac239 infection (37), suggesting that microbial translocation and intestinal integrity disruption appear in the late acute phase and last to the chronic stage in SIV-infected NPMs. Impairment in the balance between gut microbes and the host immune system could culminate in microbial translocation and the development of metabolic endotoxemia, leading to systemic inflammation and insulin-related metabolic disorder (58). Therefore, long-term microbial translocation in the relatively early infectious stage may induce profound and lasting changes in glucose metabolism in SIV-infected NPMs. Energy metabolism is a key factor supporting spermatogenesis. Long-term glucose metabolism disorder induced a series of pathological changes in the testes related to glucose metabolism, which finally induced spermatogenic dysfunction in NPMs with long-term SIVmac239 infection.

In conclusion, we found that long-term SIVmac239 infection in NPMs undermined pancreatic islet β cell function, partly represented by the significant reductions in cell counts and autophagy levels. Pancreatic islet β cell dysfunction led to glucose metabolism disorder at the whole-body level, which inhibited lactate production by Sertoli cells in the testicular tissues. As lactate is the main energy substrate for developing germ cells, energy depletion induced spermatogenic dysfunction in the NPMs following long-term SIVmac239 infection. These results imply that glucose metabolism disorder is primarily responsible for spermatogenic dysfunction in NPMs with long-term SIVmac239 infection. Therefore, timely and effective treatment to reverse and control glucose metabolism disorder may help limit spermatogenic dysfunction and metabolic disorder during HIV infection. Metformin (dimethylbiguanide), commonly used to treat type 2 diabetes, can improve gut microbiota composition, in turn reducing inflammation and risk of non-AIDS comorbidities in HIV infectors (59). Hence, combined cART and metformin treatment could be considered for patients with new HIV diagnosis to control viral replication, inflammation, and metabolic disorders. In addition, combined cART, metformin, and insulin treatment may be suitable for long-term infectors with glucose metabolism disorder to limit spermatogenic dysfunction.

## Data Availability Statement

Publicly available datasets were analyzed in this study. This data can be found here: (https://www.ncbi.nlm.nih.gov/geo/query/acc.cgi?acc=GSE44301).

## Ethics Statement

The animal study was reviewed and approved by the Ethics Committee of the Kunming Institute of Zoology, Chinese Academy of Sciences.

## Author Contributions

Y-TZ and T-ZS conceived and designed the experiments. T-ZS, M-XZ, H-DZ, X-HW, WP, and R-RT performed the experiments. T-ZS and Y-TZ analyzed the data. T-ZS and Y-TZ wrote the paper. All authors contributed to the article and approved the submitted version.

## Funding

This work was partly supported by the National Natural Science Foundation of China (U1802284, 82071847, 81971548, U1902210, and 32070181) and National Science and Technology Major Projects of Infectious Disease Funds (2017ZX10304402-002, 2017ZX10202102-001, 2018ZX10301406-003, 2018ZX10301101-002, and 2018ZX10301406-003).

## Conflict of Interest

The authors declare that the research was conducted in the absence of any commercial or financial relationships that could be construed as a potential conflict of interest.

## Publisher’s Note

All claims expressed in this article are solely those of the authors and do not necessarily represent those of their affiliated organizations, or those of the publisher, the editors and the reviewers. Any product that may be evaluated in this article, or claim that may be made by its manufacturer, is not guaranteed or endorsed by the publisher.
