# Supplementary material for: Glucose Metabolism Disorder Induces Spermatogenic Dysfunction in Northern Pig-Tailed Macaques (Macaca leonina) With Long-Term SIVmac239 Infection
Source: Front Endocrinol (Lausanne). 2021 Sep 24;12:745984. doi: 10.3389/fendo.2021.745984 (PMC8498567; doi:10.3389/fendo.2021.745984)
Supplement: Supplementary file 1 [file Table_1.docx]

**Supplementary table 1. Primers Sequence**

| Primers |  | Sequence |
| --- | --- | --- |
| IL1β | F | ATGGCAGAAGTACCTGAGCTCGCC |
|  | R | GCTTTTTTGCTGTGAGTCCCGGA |
| IL2 | F | TCAAACCTCTGGAGGAAGTGC |
|  | R | CAATGGTTGCTGTCTCATCAGC |
| IL4 | F | GGCAGTTCTACAGCCACCAT |
|  | R | TTCCTGTCGAGCCGTTTCAG |
| IL5 | F | ATCCCCACAGAAATTCCCGC |
|  | R | CAGTACCCCCTTGCACAGTT |
| IL-6 | F | AGCCAGCCACTGACCTCTTCAG |
|  | R | TTCTGCCAGTGCCTCTTTGCTG |
| IL7 | F | GGTATGTTTTTATTCCGTGCTG |
|  | R | ACTCTTTGTTGGTTGGGCTTCA |
| IL10 | F | CTGCCTCACATGCTTCGAGA |
|  | R | CTTGATGTCTGGGTCGTGGT |
| IL12 | F | ATGCCCCTGGAGAAATGGTG |
|  | R | GGCCAGCATCTCCAAACTCT |
| IL13 | F | GTACTGTGCAGCCCTGGAAT |
|  | R | AACTGGGCCACCTCGATTTT |
| IL15 | F | ACAGAAGCCAACTGGGTGAA |
|  | R | TGCAACTGGGGTGAACATCA |
| IL22 | F | GTTCCAGCCTTATATGCAGGAGG |
|  | R | GGACATTCTTCTGGATATGCAGG |
| TGFβ | F | GCCCTGGACACCAACTATTGCT |
|  | R | AGGCTCCAAATGTAGGGGCAGG |
| TNFα | F | GACAAGCCTGTAGCCCATGT |
|  | R | GTGAGGAGCACATGGTTGGA |
| IFNα | F | TGGGCTGTGATCTACCTCAAAC |
|  | R | GAGCCTTTTGGAACTGGTTGCC |
| IFNβ | F | GAGGAAATTAAGCAGCCGCA |
|  | R | AGTCTCATTCCAGCCAGTGC |
| IFNγ | F | CCAAATTGTCTCCTTTTACTTCAAACTT |
|  | R | CATCCCGTTTCTTTTTGTTGCTAT |
| GAPDH | F | GACCACAGTCCATGCCATCA |
|  | R | CATCACGCCACAGTTTCCC |
