# Supplementary material for: Glucose Metabolism Disorder Induces Spermatogenic Dysfunction in Northern Pig-Tailed Macaques (Macaca leonina) With Long-Term SIVmac239 Infection
Source: Front Endocrinol (Lausanne). 2021 Sep 24;12:745984. doi: 10.3389/fendo.2021.745984 (PMC8498567; doi:10.3389/fendo.2021.745984)
Supplement: Supplementary file 2 [file Table_2.docx]

**Supplementary table 2. Johnsen score**

| Score | Definition |
| --- | --- |
| 10 | Complete sepermatogenesis with many spermatozoa |
| 9 | Disorganized spematogenesis with many spermatozoa |
| 8 | Only a few spermatozoa (<5-10/tubule) |
| 7 | No spermatozoa, many spermatids |
| 6 | Only a few spermatids (<5-10/tubule) |
| 5 | No spermatids, many spermatocytes |
| 4 | Only a few spermatocytes (<5/tubule) |
| 3 | Only spermatogonia |
| 2 | Only Sertoli cells |
| 1 | No cells detected in tubular section |
